# Cowpea chlorotic mottle bromovirus replication proteins support template-selective RNA replication in *Saccharomyces cerevisiae*

**DOI:** 10.1371/journal.pone.0208743

**Published:** 2018-12-26

**Authors:** Bryan S. Sibert, Amanda K. Navine, Janice Pennington, Xiaofeng Wang, Paul Ahlquist

**Affiliations:** 1 Institute for Molecular Virology, University of Wisconsin-Madison, Madison, Wisconsin, United States of America; 2 Howard Hughes Medical Institute, University of Wisconsin-Madison, Madison, Wisconsin, United States of America; 3 John W. and Jeanne M. Rowe Center for Research in Virology, Morgridge Institute for Research, University of Wisconsin-Madison, Madison, Wisconsin, United States of America; Institut de Biologie Moleculaire et Cellulaire, FRANCE

## Abstract

Positive-strand RNA viruses generally assemble RNA replication complexes on rearranged host membranes. Alphaviruses, other members of the alpha-like virus superfamily, and many other positive-strand RNA viruses invaginate host membrane into vesicular RNA replication compartments, known as spherules, whose interior is connected to the cytoplasm. Brome mosaic virus (BMV) and its close relative, cowpea chlorotic mottle virus (CCMV), form spherules along the endoplasmic reticulum. BMV spherule formation and RNA replication can be fully reconstituted in *S*. *cerevisiae*, enabling many studies identifying host factors and viral interactions essential for these processes. To better define and understand the conserved, core pathways of bromovirus RNA replication, we tested the ability of CCMV to similarly support spherule formation and RNA replication in yeast. Paralleling BMV, we found that CCMV RNA replication protein 1a was the only viral factor necessary to induce spherule membrane rearrangements and to recruit the viral 2a polymerase (2a^pol^) to the endoplasmic reticulum. CCMV 1a and 2a^pol^ also replicated CCMV and BMV genomic RNA2, demonstrating core functionality of CCMV 1a and 2a^pol^ in yeast. However, while BMV and CCMV 1a/2a^pol^ strongly replicate each others’ genomic RNA3 in plants, neither supported detectable CCMV RNA3 replication in yeast. Moreover, in contrast to plant cells, in yeast CCMV 1a/2a^pol^ supported only limited replication of BMV RNA3 (<5% of that by BMV 1a/2a^pol^). In keeping with this, we found that in yeast CCMV 1a was significantly impaired in recruiting BMV or CCMV RNA3 to the replication complex. Overall, we show that many 1a and 2a^pol^ functions essential for replication complex assembly, and their ability to be reconstituted in yeast, are conserved between BMV and CCMV. However, restrictions of CCMV RNA replication in yeast reveal previously unknown 1a-linked, RNA-selective host contributions to the essential early process of recruiting viral RNA templates to the replication complex.

## Introduction

Positive-strand RNA viruses are the largest genetic class of viruses and include many important human pathogens such as the Zika, Chikungunya, MERS, SARS, and Dengue viruses. There is a significant need for the development of therapeutic treatments for these and other viruses. Broad spectrum antivirals that target highly conserved viral functions are particularly sought after. For positive-strand RNA viruses, such a universal feature and potential antiviral target is the assembly of cytoplasmic membrane-associated RNA replication complexes [1, 2]. The alphavirus-like super family of positive-strand RNA viruses includes hundreds of viruses that share significant similarities in the ultrastructure of their RNA replication complexes as well as homology among key viral enzymes, including the RNA-dependent RNA polymerase, an NTPase/helicase-like domain, and a RNA capping/methyltransferase domain [3–5].

**Fig 1:**
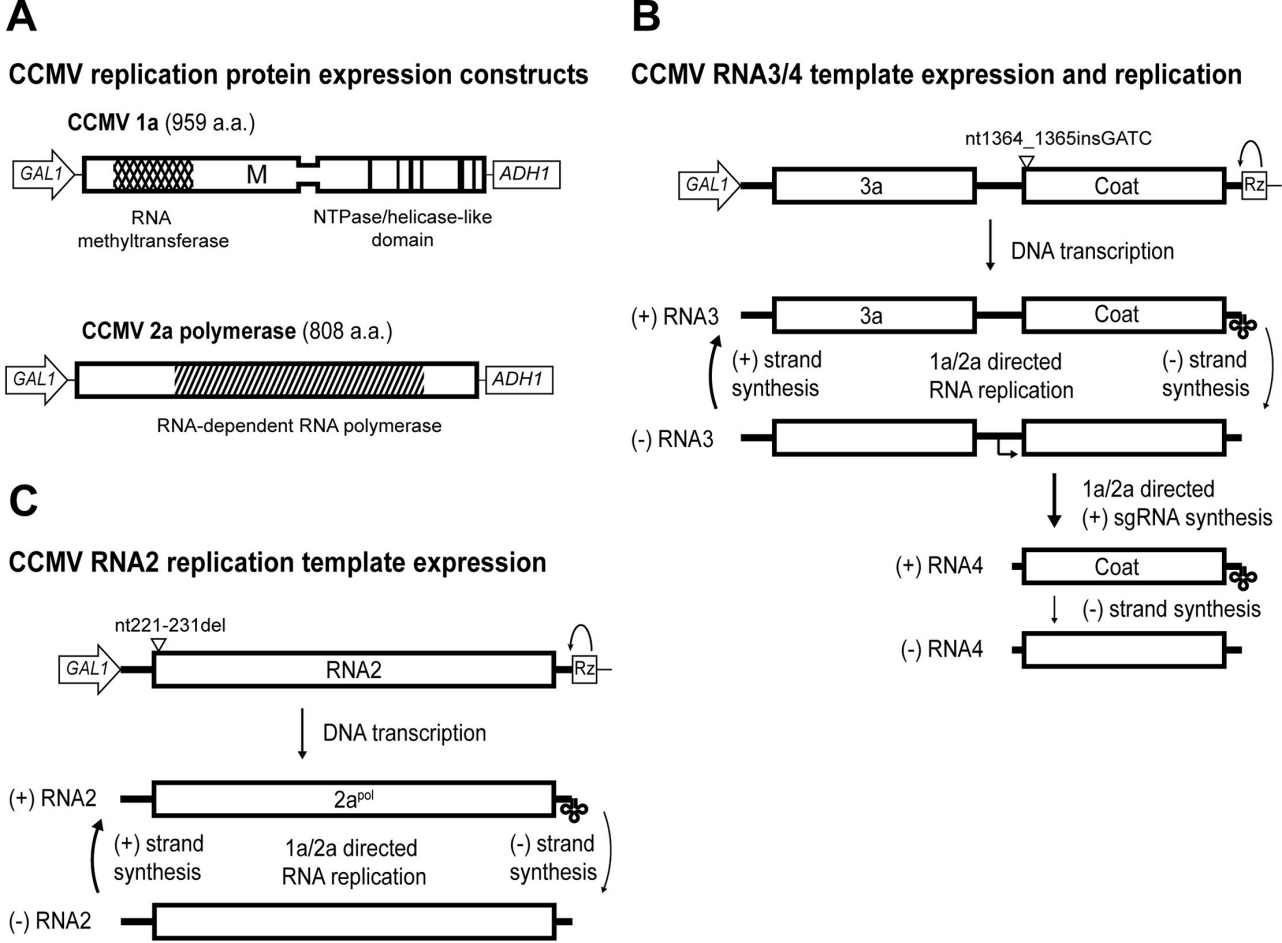
DNA launched CCMV 1a, 2a, RNA3, and RNA2 expression and replication. (A) The open reading frames for CCMV 1a and 2a from RNA1 and 2 respectively were cloned in between the yeast GAL1 promoter and the yeast ADH1 terminator. These constructs will express the viral proteins, but lack essential non-coding viral sequence required for RNA replication. Highly conserved regions of 1a responsible for methyltransferase activity (diamonds), membrane association (‘M’), and predicted helicase domains (vertical bars) are indicated. The core RNA-dependent RNA-polymerase domain of 2a^pol^ is indicated by striping. (B) Full CCMV RNA3 cDNA sequence was cloned between the GAL1 promoter and a ribozyme (Rz) derived from hepatitis delta virus that cleaves itself from the RNA leaving the natural viral 3’end. CCMV 1a and 2a^pol^ proteins direct negative-strand synthesis of RNA3, which then serves as a template for additional positive strand synthesis of both RNA3 and the subgenomic RNA4. Expression of the coat protein depends on synthesis of (+)RNA4 from the (-)RNA3 template, but is intentionally blocked in this construct by an introduced frameshift mutation. (C) Full-length CCMV RNA2 cDNA was cloned between the GAL1 promoter and ribozyme as for CCMV RNA3. A small deletion in this construct disrupts the 2a^pol^ ORF and blocks cis- expression of 2a from the template.

Cowpea chlorotic mottle virus (CCMV) is a member of the *Bromoviridae* family of viruses and has been previously studied as a model for varied replication steps within the alphavirus-like superfamily [6, 7]. CCMV systemically infects cowpea (*Vigna unguiculata*) and other dicotyledonous plants and efficiently carries out RNA replication in protoplasts from a wider range of plants, including some monocotyledons like barley (*Hordeum vulgare*) [8, 9]. CCMV has a tripartite genome and encodes four viral proteins. RNA1 encodes the multifunctional 1a protein that contains the RNA capping/methyltransferase and NTPase/helicase-like domains (Fig 1A). RNA2 encodes the RNA-dependent RNA-polymerase. RNA3 directly serves as the mRNA for a cell-to-cell movement protein, 3a, and the coat protein is translated from a subgenomic RNA4 transcribed from negative strand RNA3 (Fig 1B). RNA3/4 and their products are dispensable for intracellular RNA replication [10].

CCMV infection of plants induces dilation of and vesicular invaginations along the endoplasmic reticulum (ER) membrane that remain connected to the cytoplasm [11–13]. Similar vesicular invaginations of the ER also occur during infections of another closely related, well-studied bromovirus, brome mosaic virus (BMV) [12, 14]. For BMV, these ER invaginations, known as spherules, have been shown to be the sites of viral RNA replication [15]. RNA replication by many other viruses in and beyond the alphavirus-like superfamily occurs in similar virus-induced vesicular membrane spherules [16–18].

BMV systemically infects a range of plants and BMV RNA replication is supported in protoplasts from many plants including cowpea and barley. In such plant cells, BMV 1a + 2a^pol^ and CCMV 1a + 2a^pol^ support replication and subgenomic mRNA transcription of either BMV or CCMV RNA3 to similar levels, although heterologous 1a/2a^pol^ pairings do not support full RNA replication [8]. Many studies of CCMV and BMV in plants have productively utilized component mixing or hybrid viral proteins and RNAs to study essential mechanisms of RNA replication and viral spread [19–22].

BMV RNA replication and encapsidation can be fully reconstituted in the model yeast *S*. *cerevisiae* [23–25]. Plasmid-based expression of 1a and 2a^pol^ proteins from non-replication competent mRNAs directs positive- and negative-strand RNA synthesis from replication competent viral RNA templates provided in trans from plasmids. In plant and yeast cells, the BMV 1a protein is necessary and sufficient among viral proteins to induce spherule membrane rearrangements [15, 26]. Each BMV RNA replication complex contains highly multimerized [27, 28], membrane-associated [29, 30] 1a proteins [15]. BMV 1a also is sufficient to recruit viral RNA templates and 2a^pol^ to the replication complex [15].

The ability to carry out RNA replication in yeast has greatly facilitated many studies of BMV and other positive-strand RNA viruses [31, 32]. *S*. *cerevisiae* has a rapid cell cycle, a small and extremely well annotated genome, and many advanced tools available for genomic manipulation. Many host pathways required for BMV RNA replication have been revealed by classical yeast genetic studies as well as systematic, high throughput study of over 5,000 individual yeast gene using deletion and conditional allele expression libraries [33, 34]. Many individual steps of BMV RNA replication can be specifically assayed in yeast, including localization of 1a and 2a^pol^ proteins, membrane association of 1a, 2a^pol^, and viral RNA, 1a-induced membrane rearrangements, negative-strand RNA synthesis, positive-strand RNA synthesis, and subgenomic RNA synthesis. Yeast also support the selective encapsidation of BMV RNAs [25].

To take advantage of the benefits of the BMV / yeast system and the power of viral hybrid studies, we have now generated yeast plasmids expressing CCMV 1a, 2a^pol^ and selected genomic RNAs, and investigated their ability to support RNA replication in yeast. We show that many BMV 1a protein functions required for RNA replication complex assembly, including perinuclear localization, membrane association and rearrangements, self-interaction, and recruitment of 2a polymerase, are conserved in the related CCMV 1a in yeast. CCMV 1a and 2a^pol^ also support replication of CCMV RNA2, BMV RNA2, and BMV RNA3, although to levels lower than BMV 1a and 2a^pol^. No replication of CCMV RNA3 was detected in yeast despite the ability of both viruses to replicate CCMV RNA3 in plants. These data indicate different template-dependent host requirements for the replication of bromovirus RNAs. In the absence of BMV or CCMV 2a^pol^, CCMV 1a induced lower levels of BMV RNA3 accumulation and recruited less BMV RNA3 to a membrane-associated state than BMV 1a, indicating 1a-dependent host contributions to RNA recruitment. Further investigation of these differences will be a valuable tool to dissect essential processes such as recruiting viral RNA templates to the replication compartment.

## Results

### CCMV 1a+2a^pol^ support positive- and negative- strand replication of BMV RNA3 in yeast

To test the ability of CCMV 1a and 2a^pol^ proteins to direct RNA replication in yeast, we generated plasmids expressing CCMV 1a and 2a^pol^ mRNAs as well as a full-length CCMV RNA3 replication template (Fig 1). The plasmids expressing CCMV 1a and 2a^pol^ proteins lack viral 5' and 3' untranslated regions essential in cis for RNA replication (Fig 1A). The plasmid expressing full-length CCMV RNA3 contains a ribozyme at the 3' end to generate the authentic, non-polyadenylated viral 3' end (Fig 1B). The transcription start site of the *GAL1* promoter sequence in the plasmid has been previously shown to generate BMV RNA3 templates with the correct 5' end [35]. This expression system is identical to one previously described for BMV [15] with the exception of the viral sequences. To alleviate any possible effects on RNA3 accumulation or stability through RNA3 encapsidation by the coat protein, we introduced a frameshift mutation immediately following the initiation codon of the CCMV coat protein open reading frame. A similar mutation is present for equivalent reasons in the BMV RNA 3 replication template used in this study and most previous studies of BMV RNA3 in yeast [36].

**Fig 2:**
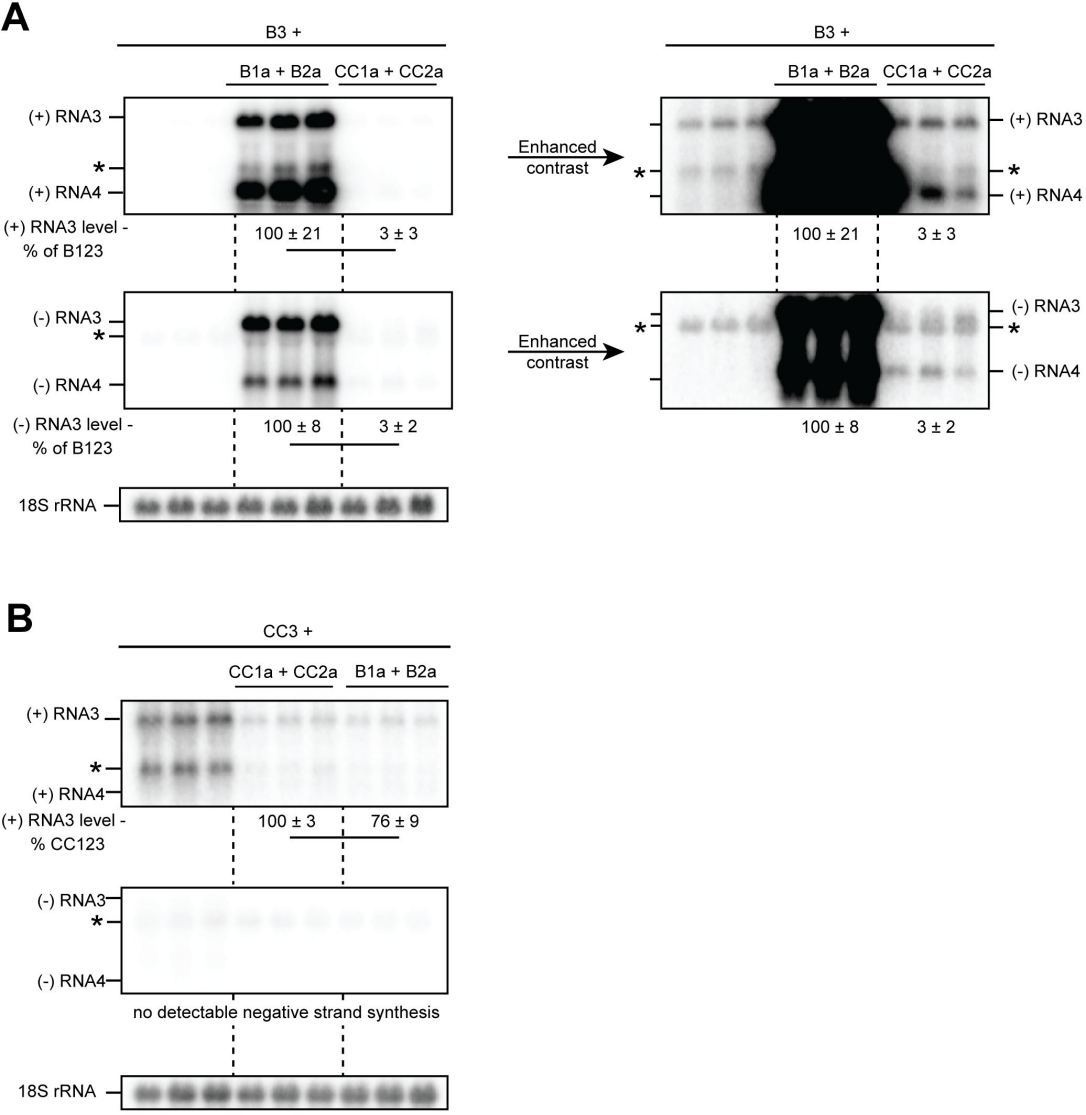
Virus and RNA template specific differences in RNA replication levels in yeast. (A) Total RNA from yeast expressing BMV RNA3 alone, lanes 1–3; BMV RNA3, BMV 1a, and BMV 2a, lanes 3–6; or BMV RNA3, CCMV 1a, and CCMV 2a, lanes 7–9 was probed for positive and negative- strand RNA3 and 4 as indicated by northern blotting. Relative levels of RNA3 are indicated beneath the blots. Blots are shown to the right with strongly enhanced contrast to better visualize replication dependent bands in lanes 7–9. 18S RNA is shown as a loading control. Bands other than (+)RNA3 present in the absence of RNA replication are marked with a an asterisk. (B) As in A, except with a CCMV RNA3 template in place of BMV RNA3. The average and standard deviation of each triplicate shown is presented beneath the blots. A line connecting two numbers indicates a statistically significant difference (p<0.05).

**Fig 3:**
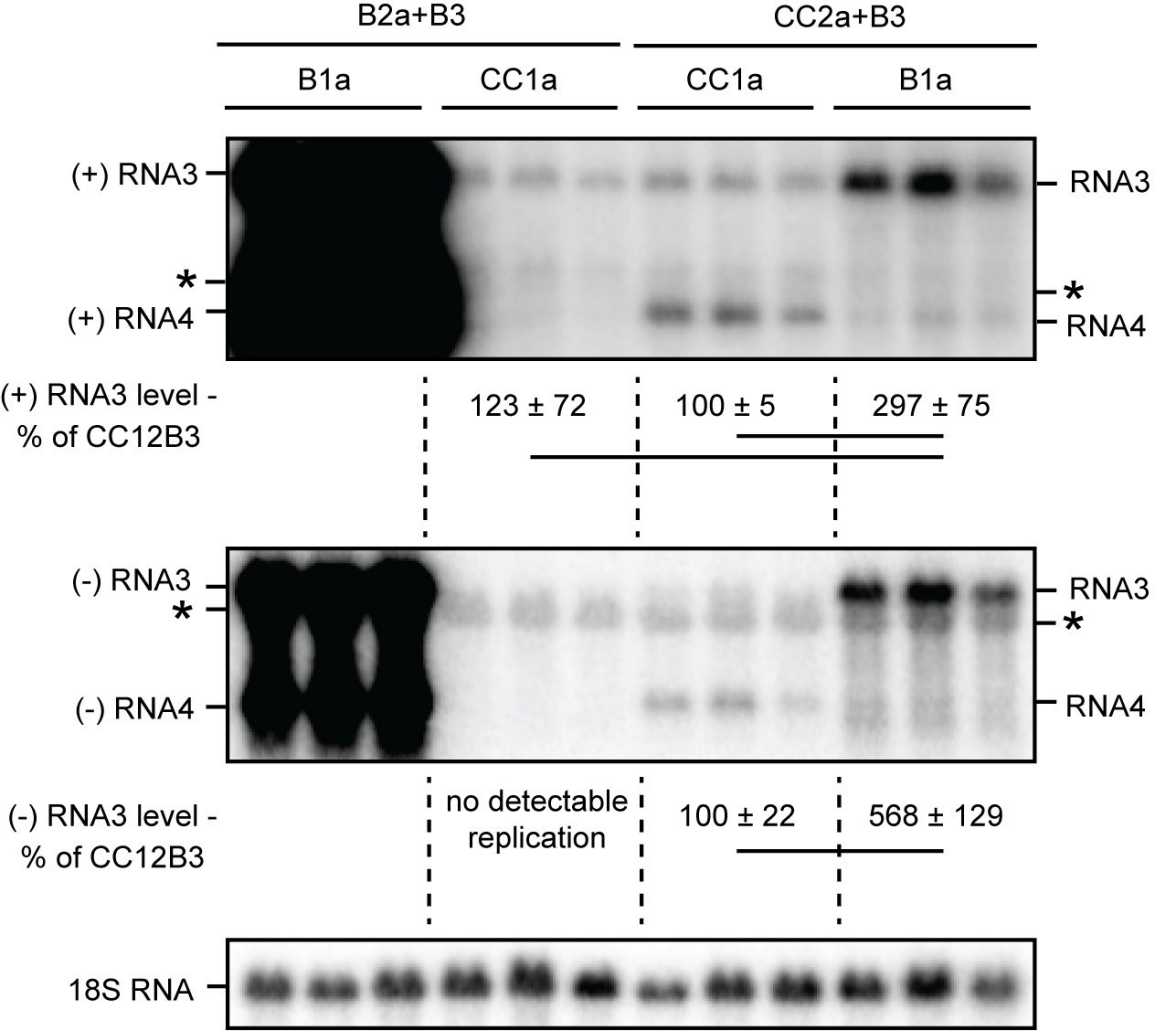
CCMV 2a polymerase is competent for negative-strand RNA synthesis in yeast. Northern blotting was used to detect viral RNA from yeast expressing BMV RNA3 with different combinations of BMV or CCMV 1a and 2a^pol^ as indicated. 18S RNA is shown as a loading control. Bands other than (+)RNA3 present in the absence of RNA replication are marked with a an asterisk. The average and standard deviation of each triplicate shown is presented beneath the blots. A line connecting two numbers indicates a statistically significant difference (p<0.05).

We first transformed yeast with plasmids expressing the BMV RNA3 replication template and either BMV 1a+2a^pol^ or CCMV 1a +2a^pol^. Consistent with previous studies, we found that BMV 1a+2a^pol^ induced robust RNA synthesis as demonstrated by increased abundance of positive-strand RNA3 as well as the presence of positive-strand RNA4 and negative-strand RNA3 and RNA4, which all require RNA replication for synthesis (Fig 2A). CCMV 1a+2a^pol^ did not substantially increase positive-strand RNA3 accumulation over RNA3 alone. However, 1a+2a dependent negative-strand RNAs and positive-strand RNA4 were detectable, as seen more clearly when the blot intensity and contrast were enhanced, indicating that 1a+2a^pol^ dependent RNA replication did occur (Fig 2A, right panels; see also Fig 3A below for further illustration). However, the levels of positive- and negative- strand RNA3 in cells expressing CCMV 1a+2a^pol^ were approximately 3% of those cells expressing BMV 1a+2a^pol^ (Fig 2A).

We then repeated the experiment using our CCMV RNA3 replication template. Notably, as determined by the absence of any 1a+2a^pol^-dependent bands on the northern blot, no replication of CCMV RNA3 was detectable in yeast with either 1a+2a^pol^ pair (Fig 2B). Additionally, we never observed an increase in positive-strand CCMV RNA3 accumulation in the presence of 1a+2a. Rather, the level of positive-strand CCMV RNA3 detected in cells co-expressing BMV or CCMV 1a+2a was less than the levels in cells expressing RNA3 alone. Since BMV 1a+2a^pol^ and CCMV 1a+2a^pol^ each replicate CCMV RNA3 to easily detectable levels in plant cells [20], our data reveal one or more yeast-specific restriction(s) on CCMV RNA3’s activity as an RNA replication template. Further, since BMV RNA3 was a functional template for 1a+2a^pol^ of both viruses in yeast, there must be differences in the host requirements between BMV and CCMV RNA3 replication.

### 1a/2a^pol^ compatibility is required in yeast as in plants

In plant cells, bromovirus RNA replication requires functional compatibility between the RNA replication proteins 1a and 2a^pol^. CCMV 1a and BMV 2a^pol^ do not support any detectable RNA synthesis in protoplasts, while BMV 1a/CCMV 2a^pol^ support negative-strand, but not positive-strand, RNA synthesis [37]. To determine if these compatibility requirements were conserved in yeast, and to separately test CCMV 1a and 2a^pol^ function, we tested the ability of heterotypic 1a/2a^pol^ pairs to direct replication of BMV RNA3. For CCMV 1a and BMV 2a^pol,^ the results were indistinguishable from RNA3 in the absence of any viral replication proteins (Fig 3; see also B1a+B2a+B3 and B3 only in Fig 2A). Thus, as in plant cells [37], CCMV 1a and BMV 2a^pol^ did not synthesize any detectable negative-strand RNA3, did not increase positive-strand RNA3 levels over the starting DNA plasmid transcripts, and did not produce any RNA4 of either polarity. In contrast, and also as in plant cells [37], BMV 1a and CCMV 2a^pol^ supported negative-strand BMV RNA3 synthesis, and intriguingly did so at levels over five-fold higher than the homologous CCMV 1a and 2a^pol^ pair (Fig 3, middle panel). BMV 1a/CCMV 2a^pol^ also increased positive-strand RNA3 levels nearly three-fold, but produced only a trace RNA4 signal. Together, these findings and the prior plant results suggest that BMV 1a/CCMV 2a^pol^ may have a general defect in positive-strand RNA synthesis, and that the increase in positive strand BMV RNA3 accumulation may primarily result from initial template recruitment by BMV 1a only, as documented in the next section.

### Template-specific differences in 1a-dependent recruitment of RNA to membrane

**Fig 4:**
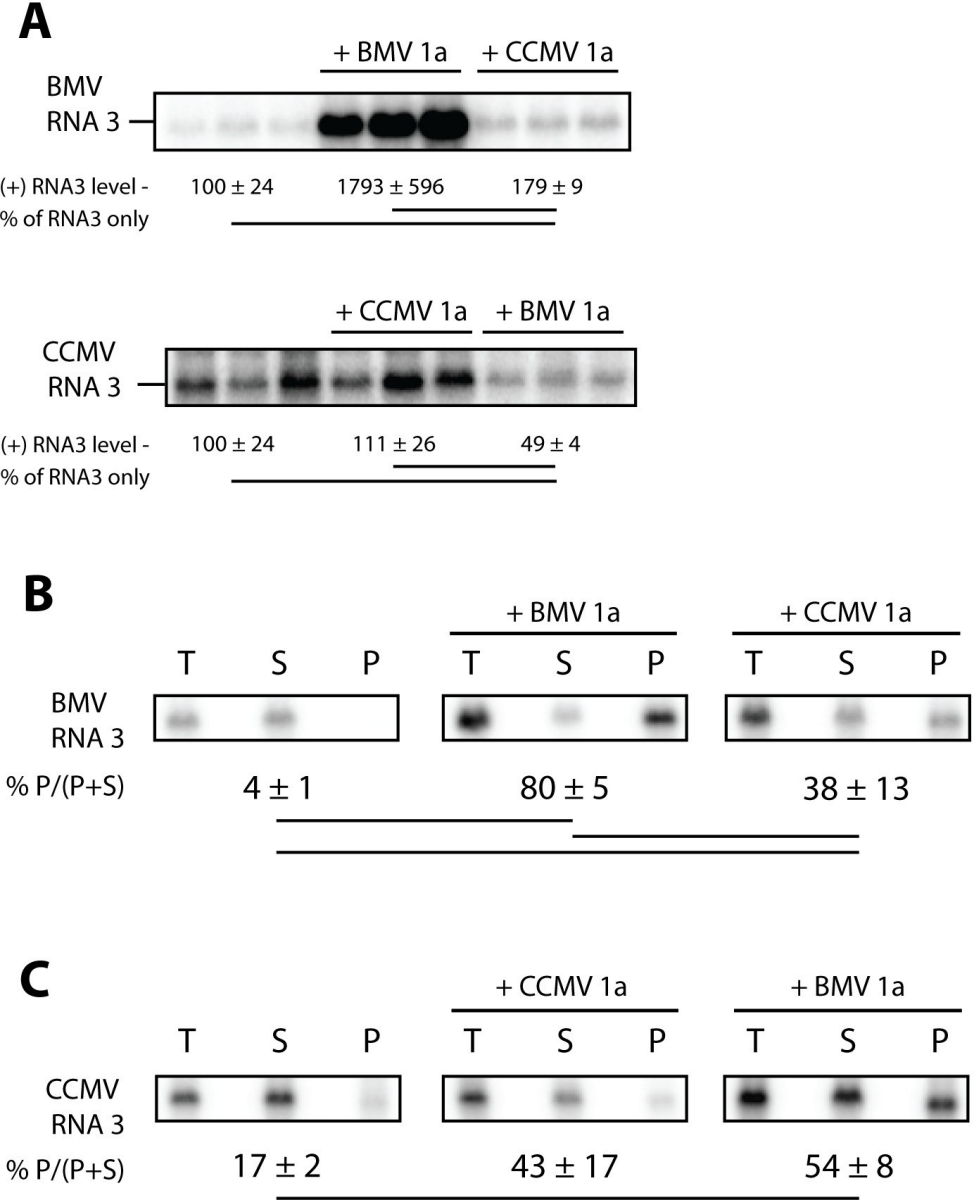
CCMV 1a is defective relative to BMV 1a in recruiting RNA templates to membranes. (A) Positive-strand BMV or CCMV RNA3 accumulation was detected by northern blotting from cells expressing RNA3 alone or with BMV or CCMV 1a as indicated. (B,C) As in (A) except yeast were lysed and centrifuged to pellet cellular membranes prior to RNA isolation. Accumulation of positive-strand RNA3 was assayed from total lysate (T), supernatant (S), and pellet (P) fractions. The relative percentage of RNA in the pellet fraction is indicated below each gel as the average and standard deviation quantified from three fractionations. A line connecting two numbers indicates a statistically significant difference (p<0.05).

An early, essential step in bromovirus RNA replication is recruitment of the RNA template to the replication complex, which is directed by viral protein 1a. Such recruitment of BMV RNA3 to the replication complex by BMV 1a markedly increases RNA3 stability and accumulation in yeast [38]. To determine if CCMV 1a also stimulates RNA3 accumulation, we measured BMV RNA3 levels in yeast expressing RNA3 alone or with BMV or CCMV 1a. Consistent with previous studies [36, 38], co-expressing BMV 1a and RNA3 stimulated BMV RNA3 accumulation over 15-fold above that for yeast expressing RNA3 alone (Fig 4A). In contrast, CCMV 1a only stimulated BMV RNA3 accumulation less than two-fold (Fig 4A). We then tested whether either 1a protein would increase CCMV RNA3 accumulation, and found no increased accumulation of CCMV RNA3 when either BMV or CCMV 1a was co-expressed (Fig 4A). Accumulation of CCMV RNA3 was consistently lower in cells co-expressing BMV 1a than in cells expressing CCMV RNA3 only (Fig 4A).

A previous study of mutations in the BMV 1a C-terminal helicase domains found that some mutated 1a proteins, which induced low or no stimulation of RNA3 accumulation, nonetheless recruited reduced amounts of BMV RNA3 to a membrane-associated state that is thought to represent a more direct assay for early association with the RNA replication complex [39]. To determine if CCMV 1a recruited viral RNAs to a membrane-associated state in yeast, we used centrifugation to fractionate detergent-free yeast lysates into membrane-depleted supernatant and membrane-enriched pellet fractions [39]. As expected, ~80% of BMV RNA3 was found in the pellet fraction when co-expressed with BMV 1a, as opposed to 4% when expressed alone (Fig 4B). Interestingly, co-expressing CCMV 1a and BMV RNA3 also increased the percentage of RNA in the pellet fraction to ~38% (Fig 4B). Co-expressing either BMV or CCMV 1a protein with CCMV RNA3 also increased the percentage of RNA in the pellet fraction from ~17% to ~50% (Fig 4C).

### CCMV 1a+2a^pol^ replicate CCMV RNA2 in yeast

**Fig 5:**
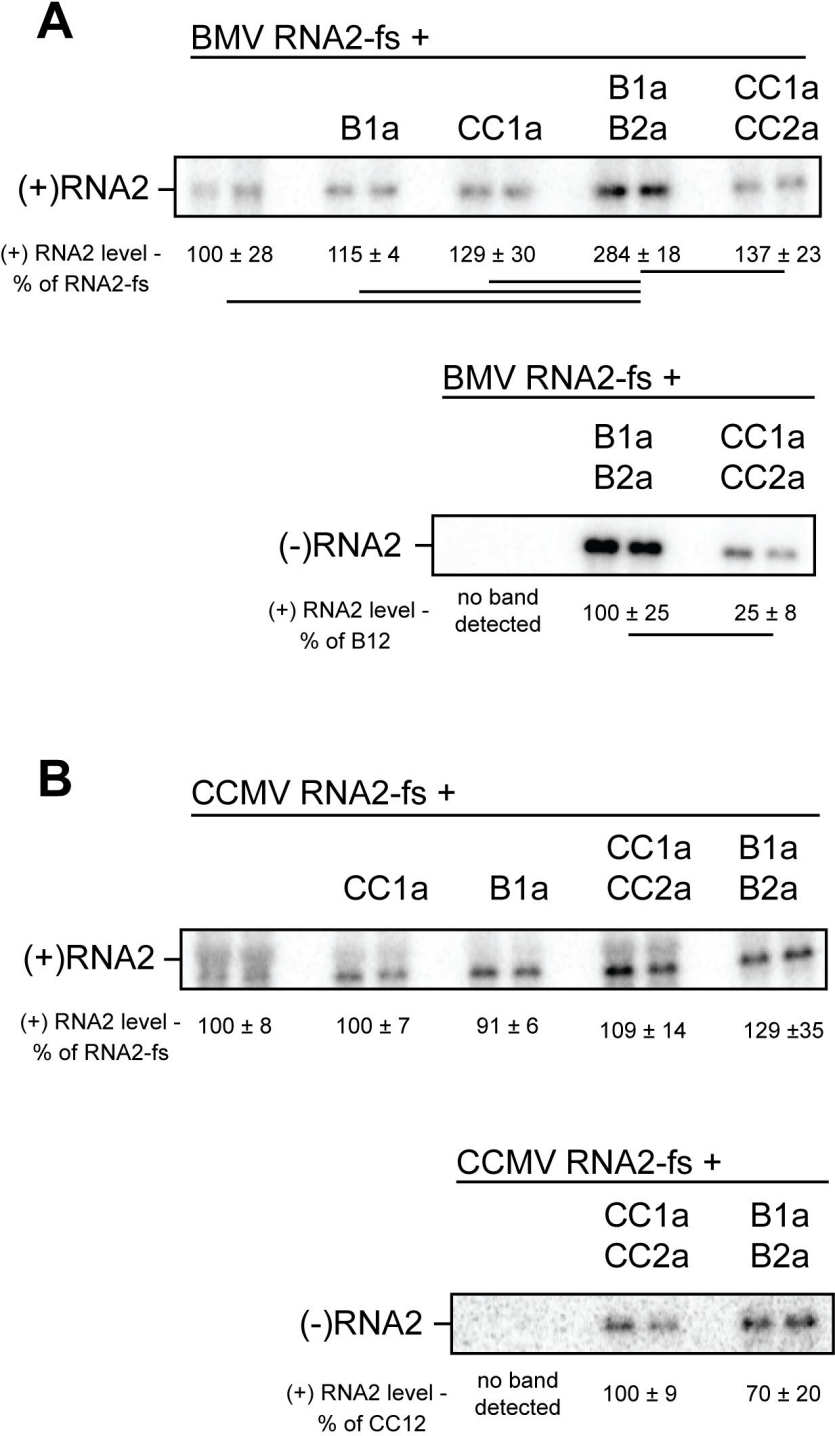
1a-dependent RNA accumulation and replication of bromovirus RNA2 templates in yeast. (A) Northern blot from total RNA from yeast expressing BMV RNA2 alone or with BMV 1a and/or 2a or CCMV 1a and/or 2a as indicated. Relative levels of RNA2 are indicated beneath the blots. (B) As in (A) except with a CCMV RNA2 template in place of BMV RNA3. The average and standard deviation of three replicates is shown beneath the lanes. A line connecting two numbers indicates a statistically significant difference (p<0.05).

Given the differences in replication and recruitment between BMV and CCMV RNA3 templates, we next tested RNA2 templates from both viruses in order to further characterize the function of CCMV 1a+2a^pol^ in yeast. BMV RNA2 is replicated by BMV 1a+2a^pol^ in yeast, although little increase in accumulation of positive-strand RNA2 was observed upon co-expressing BMV 1a [40]. We used an RNA2 template in which 2a expression is blocked by a frameshift mutation to eliminate any possible effects of excess 2a^pol^ protein or cis-interaction between 2a^pol^ and RNA2. Our results were consistent with previous findings [40], with BMV 1a increasing BMV RNA2 levels by only 15%, but BMV 1a+2a^pol^ increasing positive-strand RNA2 levels 184% over RNA2 alone (Fig 5A). Negative-strand BMV RNA2 was synthesized by both BMV 1a+2a^pol^ and CCMV 1a+2a^pol^, with CCMV 1a+2a^pol^ synthesizing 25% as much negative-strand BMV RNA2 as BMV 1a+2a^pol^ (Fig 5A, lower blot). Co-expressing CCMV 1a+2a^pol^ increased positive-strand BMV RNA2 accumulation by 37%. This level is lower than the 184% increase from BMV 1a+2a^pol^ and only slightly higher than the 29% increase from CCMV 1a alone. In total, the levels of RNA replication of BMV RNA2 by CCMV 1a+2a^pol^ were much closer to those of BMV 1a+2a^pol^ than for the BMV RNA3 template (Figs 2A and 5A).

Unlike the other RNA templates tested in this work thus far, CCMV RNA2 has not been shown to be replicated by the heterologous BMV 1a+2a^pol^ in plant cells. In fact, CCMV RNA2 replication was not detected in barley protoplasts co-transfected with BMV RNAs 1, 2, and 3 [21]. To test CCMV RNA2 replication in yeast, we generated a plasmid expressing CCMV RNA2 with a frameshift mutation blocking 2a expression, similar to the BMV RNA2 template used above. Although neither BMV nor CCMV 1a alone changed accumulation of this plasmid-derived CCMV RNA2 transcript, co-expressing either BMV or CCMV 1a+2a^pol^ yielded synthesis of negative-strand CCMV RNA2, with somewhat higher levels produced by CCMV 1a+2a^pol^ (Fig 5B). Thus, CCMV 1a+2a^pol^ not only are functional in yeast for synthesizing negative-strand CCMV RNA2, but for this template are slightly more active than their BMV counterparts. Despite this activity, neither BMV nor CCMV 1a+2a^pol^ significantly elevated positive-strand CCMV RNA2 over its levels with no viral protein or either 1a protein alone (Fig 5B), implying defects in positive-strand CCMV RNA2 synthesis.

**Fig 6:**
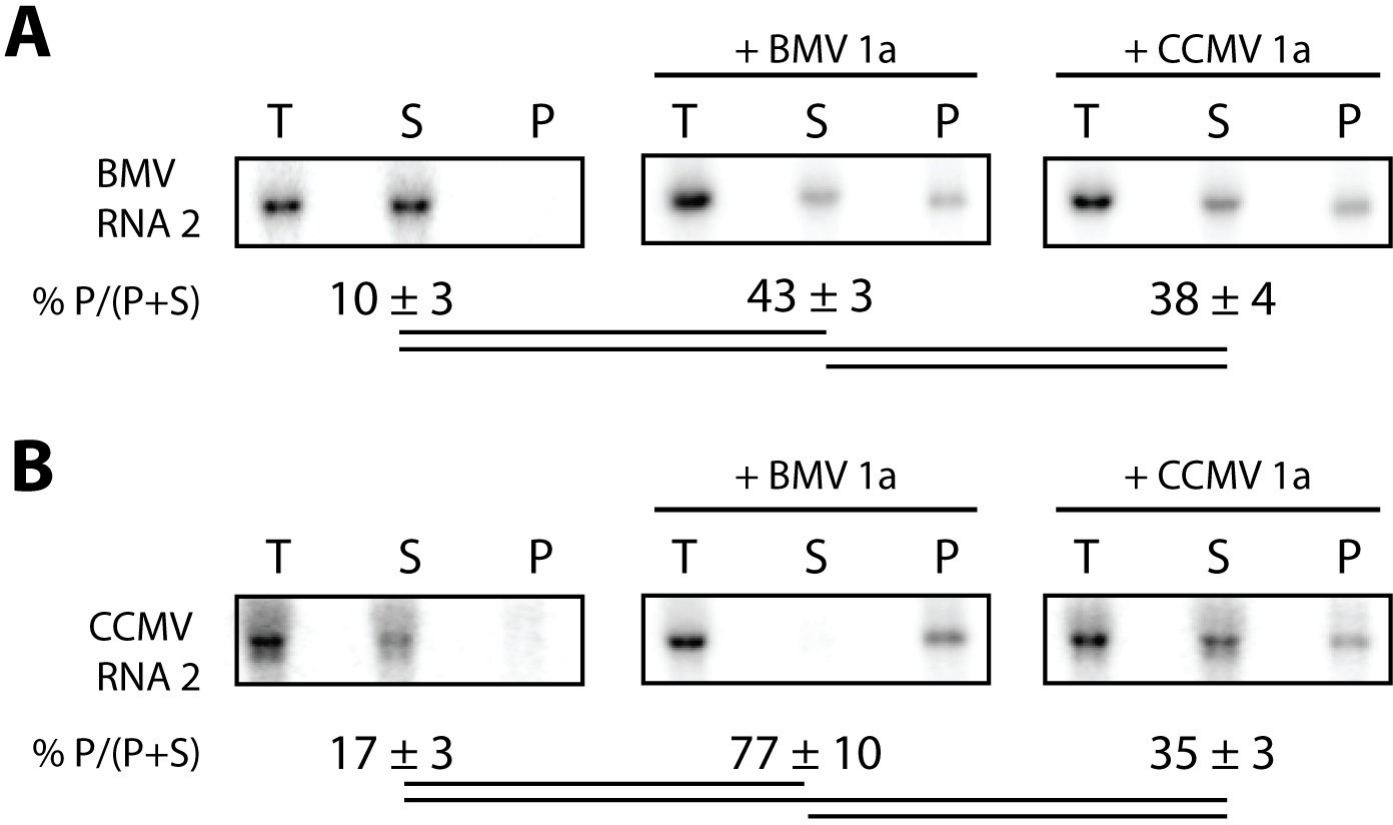
CCMV 1a recruits RNA2 templates to membranes. (A) Cells expressing BMV or CCMV RNA2 alone or with BMV or CCMV 1a as indicated were lysed and centrifuged to pellet cellular membranes prior to RNA isolation. Accumulation of positive-strand RNA2 was assayed by northern blotting from total lysate (T), supernatant (S), and pellet (P) fractions. The relative percentage of RNA in the pellet fraction is indicated below each gel as the average and standard deviation quantified from three fractionations. A line connecting two numbers indicates a statistically significant difference (p<0.05).

Since significant stimulation of positive-strand RNA template accumulation was not observed for any combination of 1a/RNA2, yet both homologous 1a+2a^pol^ combinations synthesized negative-strand products from these RNA2 templates, we further examined recruitment of these RNAs to the replication complex by testing their membrane association, as done in Fig 4BC for the RNA3 templates. As expected from the negative-strand synthesis results, both BMV and CCMV 1a proteins recruited BMV and CCMV RNA2 to membranes. Similar fractions of BMV RNA2 were recruited to membranes by BMV and CCMV 1a at 43% and 38%, respectively, compared to 10% for BMV RNA2 alone (Fig 6A). The highest percentage of membrane-associated RNA was observed for BMV 1a and CCMV RNA2 at 77%, while only 35% of CCMV RNA2 was membrane-associated when co-expressed with CCMV 1a (Fig 6B).

### CCMV 1a and 2a^pol^ replication proteins localize to ER in yeast

**Fig 7:**
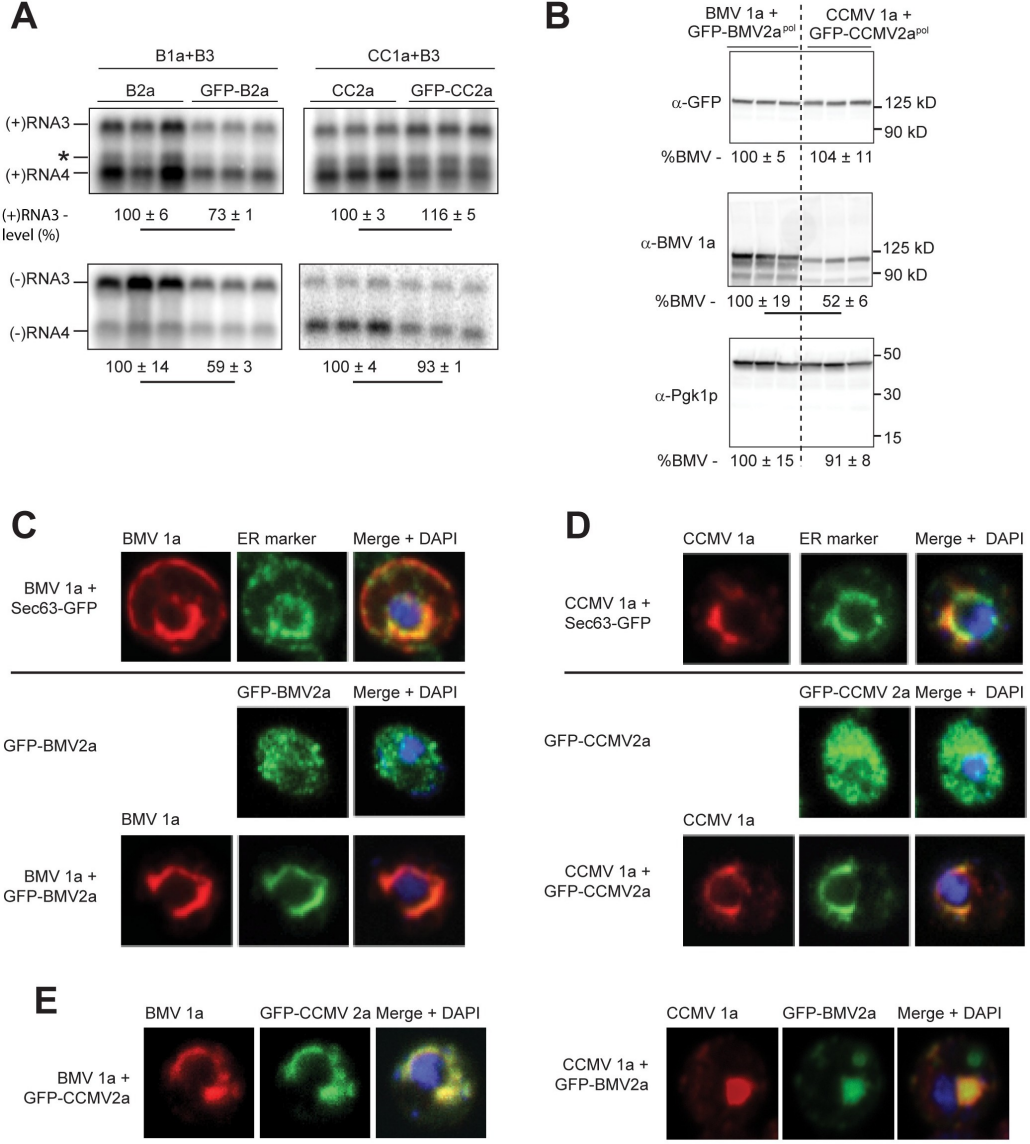
CCMV 1a localizes to the perinuclear ER and recruits CCMV 2a. (A) Replication of BMV RNA3 by BMV and CCMV with untagged 2a and GFP-2a was detected by northern blotting. (B) Western blotting was performed on total protein lysate from yeast expressing BMV 1a and GFP-BMV 2a or CCMV 1a and GFP-CCMV 2a using the antibodies indicated. Pgk1p is an endogenous yeast protein used as a loading control. The average and standard deviation of three replicates is shown beneath the lanes. A line connecting two numbers indicates a statistically significant difference (p<0.05). (C) Fluorescence confocal microscopy was used to image cells expressing BMV 1a (red) and Sec63-GFP (ER marker), BMV GFP-2a (green) only, or BMV 1a and BMV GFP-2a. DNA was stained with DAPI as a nuclear marker. (D,E) As for (B) with (D) CCMV 1a and CCMV GFP-2a or (E) the heterologous 1a/2a combinations as indicated. All images are projections of a confocal z-stack.

Given the differences that we observed between CCMV and BMV 1a and 1a+2a^pol^ in RNA replication and recruitment, we tested other 1a and 2a^pol^ functions in yeast. Accordingly, we first used western blotting to compare BMV and CCMV 1a and 2a^pol^ accumulation in yeast. To visualize 2a^pol^ protein accumulation and localization (see below), we used N-terminal GFP fusions. As in a previous report [41], GFP-BMV2a^pol^ showed function near that of wildtype BMV 2a^pol^ in replicating BMV RNA3 (Fig 7A). GFP-CCMV2a^pol^ showed wildtype functionality in replicating BMV RNA3 (Fig 7A), demonstrating again that GFP fusion did not inhibit 2a^pol^ function. Western blotting for GFP confirmed that GFP-BMV2a and GFP-CCMV2a were expressed to similar levels (Fig 7B). BMV and CCMV 1a were detected with a polyclonal antibody against an N-terminal BMV 1a fragment [42]. The signal for CCMV 1a was only 52% of BMV 1a. However, this difference is most likely primarily due to a lower affinity of the polyclonal antibody for CCMV than BMV, as it was raised against BMV 1a a.a. 1–515, which has only 74% amino acid identity with the corresponding region of CCMV 1a.

Next we used confocal microscopy to check for proper localization of the 1a and 2a^pol^ proteins in yeast as a pre-requisite for RNA replication complex assembly. As noted in the introduction, BMV and CCMV RNA replication in plant cells occurs in spherular invaginations of ER membranes, primarily on the perinuclear ER [11, 42]. When expressed in the absence of 2a^pol^, BMV and CCMV 1a proteins each localized to the perinuclear ER membranes, colocalizing strongly with a Sec63-GFP ER marker (Average Mander’s coefficient over five representative cells each for BMV1a:Sec63-GFP = 0.87 ± 0.08, and for CCMV1a:Sec63-GFP = 0.79 ± 0.07.) (Fig 7C and 7D, top panels). When expressed in the absence of 1a, BMV and CCMV 2a^pol^ each showed a diffuse distribution throughout the cytoplasm (Fig 7C and 7D, middle panels). When co-expressed with BMV 1a, the majority of BMV 2a^pol^ was recruited to the perinuclear ER, as in prior reports [41, 43]. Similarly, CCMV 1a recruited most of GFP-tagged CCMV 2a^pol^ to the perinuclear ER. Thus, the ability of the 1a protein to localize to the perinuclear ER and to recruit 2a^pol^ polymerase to these membranes, including in yeast cells, is conserved between BMV and CCMV.

Consistent with our observation that BMV 1a+CCMV 2a support negative-strand RNA synthesis (Fig 3), BMV 1a recruited GFP-CCMV 2a to the perinuclear ER (Fig 7E, left panel). In all cells, for all combinations of 1a and 2a, 1a and 2a colocalized at sites adjacent to the nucleus. For BMV 1a+GFP-CCMV 2a, as well as BMV 1a+GFP-BMV 2a and CCMV 1a+GFP-CCMV 2a, > 70% of cells displayed a ‘halo-like’ perinuclear localization with a contiguous layer of 1a surrounding at least 50% of the nuclear perimeter. In contrast, such a nuclear halo distribution was observed in < 40% of cells expressing the non-functional combination of CCMV 1a and GFP-BMV 2a. The remaining > 60% of cells expressing CCMV 1a-GFP-BMV 2a exhibited one or more large, globular punctae of these proteins (Fig 7E, right panel).

### CCMV 1a is sufficient to induce ER membrane rearrangement in yeast

**Fig 8:**
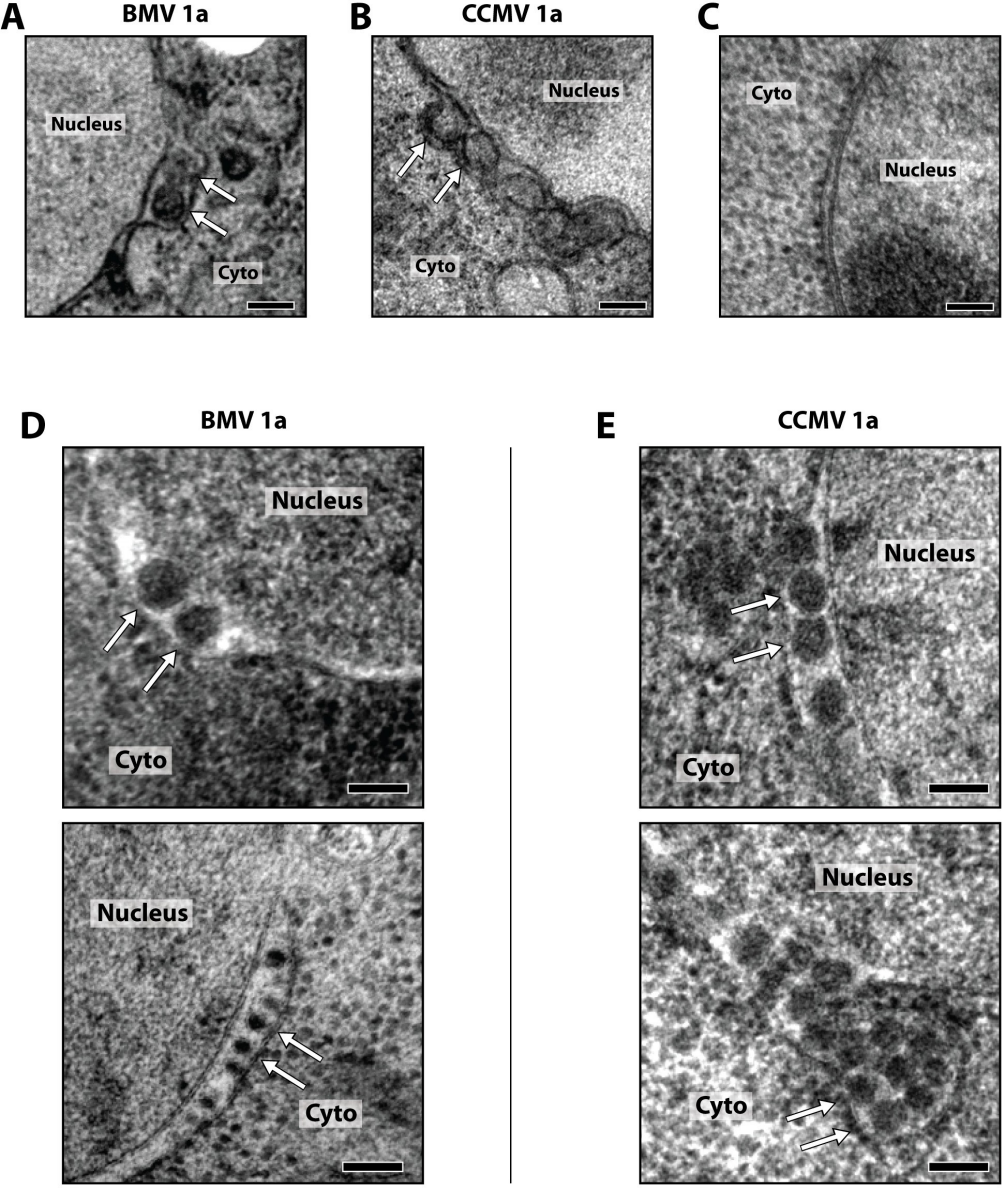
CCMV 1a is sufficient to induce spherule membrane rearrangements along the ER. Yeast expressing BMV 1a (A) or CCMV 1a (B) were prepared for EM using conventional chemical fixation and embedding (see Methods for details). Arrows identify 1a protein induced invaginations along the perinuclear ER. (C, D, E) Representative images of yeast expressing only the non-coding BMV RNA3 template (C) or BMV 1a (D) or CCMV 1a (E) prepared by high-pressure freezing and freeze substitution. All scale bars are 100 nm.

To determine if CCMV 1a induces any membrane rearrangements in yeast, we performed transmission electron microscopy of yeast expressing BMV 1a and CCMV 1a. The yeast were chemically fixed and prepared following protocols previously described to visualize BMV 1a induced membrane rearrangements [15]. We observed vesicular structures in the dilated perinuclear ER in cells expressing either BMV 1a (Fig 8A) or CCMV 1a (Fig 8B). The vesicles were typically in the range of 60-80nm in diameter and largely indistinguishable between the two 1a proteins. These yeast results match the prior demonstration that CCMV induces such spherule invaginations on ER membranes in its natural plant hosts [11–13] and extensive prior demonstrations that such spherules are the sites of RNA genome replication for other bromoviruses [12, 14, 15] and for many other alphavirus-like viruses [16–18]. As previously reported [15, 44], no such structures or dilation of the perinuclear ER lumen were observed in yeast lacking BMV components or expressing only the non-coding BMV RNA3 template.

In other studies of viral replication complexes, fixation of cells by high pressure freezing and freeze substitution (HPF) was found to allow better visualization of membrane rearrangements [45, 46]. The extremely rapid fixation of the sample followed by freeze-substitution and embedding greatly reduces fixation and dehydration artifacts associated with traditional chemical fixation [47].

Using HPF to examine yeast cells expressing BMV or CCMV 1a, we again observed typical spherular vesicles in the lumen of the perinuclear ER membrane (Fig 8D and 8E). No membrane rearrangements were observed in yeast expressing only the non-coding BMV RNA3 template (Fig 8C). The overall morphology of spherules observed in HPF-fixed cells was very similar to that observed in chemically fixed cells. As expected, the increased preservation of the dense yeast cytoplasm in HPF-fixed cells resulted in lower contrast of the membranes relative to chemically fixed cells, however the membrane was still clearly visible in many instances. In HPF fixed cells, for both BMV and CCMV 1a, occasional cells displayed a greater frequency of smaller diameter spherules (Fig 8D, bottom), and these cells generally also displayed larger numbers of spherules. The implication that HPF may preserve some 1a-induced membrane rearrangements lost in chemical fixation, and related points, are considered further in the Discussion.

## Discussion

Despite ongoing advances in understanding critical mechanisms of positive-strand RNA virus RNA replication complex assembly and function, many questions remain. Some important insights into aspects of these processes, including host dependencies, have been obtained through studies of BMV [31, 48, 49], flock house nodavirus [50, 51], and tombusvirus [32, 52] RNA replication in the model organism *S*. *cerevisiae*. Here we examined RNA replication and replication complex assembly of another bromovirus, CCMV, in yeast to better define and understand conserved features of bromovirus replication, and to develop further tools to study these processes. Our results show that CCMV RNA replication protein 1a parallels BMV 1a in inducing spherule membrane rearrangements along the perinuclear ER (Fig 8B and 8D), recruiting CCMV 2a^pol^ and RNA templates to these replication complexes (Figs 4–7), and supporting negative- and positive-strand RNA synthesis for multiple bromovirus RNAs (Figs 2 and 3). However, despite this significant conservation of function, CCMV 1a+2a^pol^-driven replication of BMV RNA3 in yeast cells was dramatically lower relative to BMV 1a+2a^pol^ (Fig 2A) than has been previously observed in plants [8, 20]. Additionally, CCMV RNA3 failed to function detectably as a template for either CCMV or BMV RNA replication proteins in yeast (Fig 2B). These findings reveal new host dependencies in several distinguishable steps of bromovirus RNA replication, as discussed further below.

### CCMV 1a induces ER membrane spherules and recruits CCMV 2a^pol^ in yeast

Prior work showed that BMV 1a is the only viral factor required to induce membrane rearrangements in plants and yeast [15, 26]. Moreover, the enzymatic activities of 1a, including RNA capping functions [53] and NTPase activity [36] are not required for such spherule formation [39]. Other positive-strand RNA viruses including flock house nodavirus and the alphavirus Semliki Forest virus (SFV) have been shown to require viral RNA-dependent RNA synthesis to induce membrane rearrangement [54, 55]. Interestingly, though, expressing partially uncleaved SFV replicase in the absence of a viral RNA template induces spherule-like invaginations similar to those induced during infection [56].

We show here that CCMV 1a, like BMV 1a, induces spherular ER membrane invaginations in yeast (Fig 8B and 8E) in the absence of other viral factors. Though BMV and CCMV are related, their 1a proteins share only 68% identity at the amino acid level and the viruses share few systemic hosts. Moreover, there is no evolutionary pressure for either virus to function in yeast, so the observation of common phenotypes between these two fairly divergent proteins suggests that 1a-induced ER membrane rearrangement in yeast occurs through 1a functions that are conserved due to being essential in natural BMV and CCMV infections. When co-expressed in yeast, CCMV 1a & 2a colocalize to the perinuclear ER (Fig 7D), suggesting that the ER is the likely site of CCMV RNA replication. However, further studies will be required to conclusively test this and in particular whether the spherule interior is the site of CCMV RNA replication, as has been shown for BMV [15].

Numerous host factors required for proper BMV 1a membrane rearrangement have been identified through yeast classical and molecular genetics. These include membrane remodeling proteins such as the reticulon homology domain proteins, members of the endosomal sorting complex (ESCRT), and components of coat protein complex II (COPII), as well as an acyl-coA binding protein involved in lipid synthesis [57–60]. Additional host factors are dispensable for spherule formation but required for viral RNA recruitment and/or replication, including the RNA binding Lsm1 protein and Δ9 fatty acid desaturase [61, 62]. Building on our results here, future studies may compare the effects of these and other host proteins on BMV and CCMV 1a membrane rearrangements to better understand their mechanism and identify well-conserved, essential interactions.

In plant and yeast cells, BMV 1a recruits BMV 2a^pol^ to ER membranes by interaction of 1a’s NTPase/helicase-like domain with an N-terminal 2a^pol^ region preceding the polymerase domain [41, 63]. Similarly, we found in yeast that BMV 1a and CCMV 1a each recruit CCMV 2a^pol^ from a diffuse cytoplasmic distribution to the ER membrane (Fig 7D and 7E). Intriguingly, however, CCMV 1a’s normal halo-like perinuclear ER localization (Fig 7D) was deranged into large globular structures by co-expressing BMV 2a^pol^ (Fig 7E). BMV 2a^pol^ was previously found to alter BMV 1a-induced ER membrane rearrangements from spherule invaginations to large multi-layer membrane stacks encircling much of the nucleus [44]. However, BMV 2a^pol^‘s effect on BMV 1a required substantial overexpression, and produced a clearly distinct, non-globular membrane morphology that was a highly active RNA replication complex [44], while the CCMV 1a + BMV 2a^pol^ globules (Fig 7E) had no detectable function in RNA synthesis (Fig 3). Nevertheless, the ability of BMV 2a^pol^ to switch 1a-dependent membrane structures into two distinguishable alternate forms appears likely to provide useful insights into 1a-membrane interactions, 1a-2a^pol^ interactions, and likely 1a-1a interactions.

### CCMV 1a, RNA3 and possibly 2a^pol^ have replication defects in yeast

In yeast, in addition to inducing typical RNA replication vesicles similar in location, morphology and abundance to BMV 1a, CCMV 1a+2a^pol^ replicated both BMV and CCMV RNA2 to levels similar to that by BMV 1a+2a^pol^ (Fig 5). However, CCMV 1a+2a^pol^ replication of BMV RNA3 was over 30-fold lower than for BMV 1a+2a^pol^ (Fig 2A). This is in contrast to multiple demonstrations that the two viruses replicate BMV RNA3 to similar levels in plant cells [8, 20, 37]. Thus, relative to plant cells, in yeast there must be one or more restrictions of CCMV 1a or 2a^pol^ function for BMV RNA3 templates.

While the requirement for 1a/2a^pol^ compatibility for RNA synthesis makes it challenging to assign some restrictions specifically to 1a, 2a^pol^, or both, our data clearly shows yeast-specific defects in certain CCMV 1a functions. First, the different replication levels of BMV RNA3 by CCMV 1a+CCMV 2a^pol^ and BMV 1a+CCMV 2a^pol^ (Fig 3) must be due to differences in 1a function or interaction, since the 2a^pol^ and RNA template are identical between those conditions. Additionally, in the absence of 2a^pol^, CCMV 1a induced dramatically less RNA accumulation and membrane association of BMV RNA3, measures of RNA recruitment to the replication complex, than BMV 1a (Fig 4A and 4B). CCMV 2a^pol^ synthesized negative-strand BMV RNA3, particularly in the presence of BMV 1a (Fig 3), but we cannot rule out yeast-specific defects in other CCMV 2a^pol^ functions or interactions.

The varying activities of CCMV 1a+2a^pol^ in yeast for replicating BMV RNA2, CCMV RNA2, BMV RNA3, and CCMV RNA3 show that unique requirements can exist for the replication of different genomic RNAs even within the same virus. Among other potentially important considerations, these results suggest that prior genetic screens of BMV RNA3 replication by BMV 1a+2a^pol^ [33, 34] may have failed to identify host genes specifically required for replication of RNAs 1 and/or 2.

In yeast, while BMV RNA3 was replicated strongly by BMV 1a+2a^pol^ and weakly by CCMV 1a+2a^pol^, CCMV RNA3 was not replicated detectably by either (Fig 2B). In addition to this lack of replication, the unusual decrease of CCMV RNA3 levels in the presence of BMV or CCMV 1a+2a (Fig 2B) suggests that, in yeast, CCMV RNA3 is subject to inhibitory interactions or processes that the other CCMV and BMV RNAs tested lack or escape. The lack of BMV 1a-stimulated accumulation of CCMV RNA3 in yeast compared to BMV RNA3 (Fig 4A) shows that the barrier(s) to replication include a block at or before the essential early step of recruiting the RNA template into the replication complex. This CCMV RNA3-specific block in yeast might be related to dependence on an alternate template recognition pathway, since CCMV RNA3 lacks a conserved tRNA TΨC loop sequence [7] that is part of the required template recognition sequences of yeast-compatible RNA replication templates BMV RNA2 and RNA3 [38, 40], and is similarly present in CCMV RNA2 [7].

Overall, our data shows that many critical functions of BMV 1a and CCMV 1a are largely conserved and that, as for BMV, many CCMV functions can be reconstituted and studied in yeast. Observed restrictions of CCMV 1a, RNA, and possibly 2a^pol^ function in yeast reveal important host contributions to RNA replication and the expression of CCMV in yeast provides new tools to study the host factors required for bromovirus replication. Comparative analysis of the replication complexes of these two systems should prove a valuable tool as we learn more about replication complex structure and assembly.

## Materials and methods

### Yeast and plasmids

*S*. *cerevisiae* strain YPH500 and culture conditions were as described previously [23, 62]. BMV 1a, 2a^pol^, and RNA3 were expressed from pB1YT3, pB2YT5, and pB3MS82 [15, 36, 41]. Standard molecular cloning techniques were used to replace the BMV derived sequences in these plasmids with the corresponding CCMV sequences from pCC1TP1, pCC2TP2, and pCC3TP10 [20] to generate pCC1GCU, pCC2GCL, and pCC3GCW10 respectively. To construct plasmid pCC3-fs, a four nucleotide insertion was introduced at the SalI restriction enzyme site immediately following the first codon of the CCMV coat protein open reading frame in pCC3GCW10. This four nucleotide insertion (GTC**GATC**GAC) results in a frameshift and subsequent stop codon twenty amino acids into the open reading frame. GFP from GFP-BMV2a^pol^ (pB2YT5-G2 [41]) was cloned into pCC2GCL to generate GFP-CCMV2a^pol^ (pCC2GCL-G). Sec63-GFP was expressed from pJK59 [64]. In experiments containing frame-shifted BMV RNA2 (pB2NR3-M1[40]), BMV 1a and 2a^pol^ were expressed from pB1YT3H[36] and pB2YT3 (Ura^+^ derivative of pB2YT5). Corresponding CCMV 1a and 2a^pol^ plasmids pCC1GCH and pCC2GCU were generated as described above. A plasmid expressing a frame-shifted CCMV RNA2 template (pCow2C) was created by replacing BMV RNA2 in pB2NR3-M1 with CCMV RNA2 sequence from pCC2TP2. Nucleotides 221–231 were deleted, removing the ClaI site, resulting in a frame-shift and introducing a stop codon at that position (amino acid 38).

### Cell fractionation

Cell fractionation was done as described previously [40]. Briefly, yeast were spheroplasted and osmotically lysed. Half of the lysate was set aside for total RNA. The remaining lysate was centrifuged at 20,000 xg for 5 minutes at 4°C. The supernatant was saved as the soluble fraction and the pellet was washed once more with lysis buffer. All three fractions were adjusted to equal volume with lysis buffer and an equal volume of each fraction was used for analysis, equivalent to 2.0 μg of total RNA.

### RNA and protein analysis

Total yeast RNA was harvested from whole cells or fractionated lysate using hot phenol [65]. RNA electrophoresis and northern blotting was done as described previously [41]. Briefly, 2.0 μg of total RNA was electrophoresed through a 1% agarose-formaldehyde gel and transferred onto nylon membrane (Nytran SPC, GE Healthcare) [66]. The membranes were probed with in-vitro transcribed ^32^P-labelled RNA probes and the radioactivity was measured using storage phosphor screens imaged on a Typhoon scanner (GE Healthcare). Viral RNA isolated from yeast was detected using strand-specific probes complementary to BMV RNA3/4 nt 1250–1755 [36], CCMV RNA3/4 nt 1355–1868, BMV RNA2 nt 2572–2865 or CCMV RNA2 nt 2534–2770. The 18S rRNA probe was transcribed from pTRI RNA 18S Antisense Control Template (Ambion). Yeast total protein was isolated by bead beating in yeast lysis buffer (50 mM Tris-HCl pH 8.0, 10 mM MgCl_2_, 1mM EGTA, 2 mM EDTA, 15 mM, protease inhibitor cocktail (Sigma)). The resulting lysate was mixed with an equal volume of SDS lysis buffer (2% SDS, 90 mM HEPES pH 7.5, 30 mM DTT), boiled for 10 minutes, centrifuged at 20,000xg for 3 minutes, and the supernatant was recovered for analysis by SDS/PAGE and western blotting. A polyclonal rabbit antibody raised against an N-terminal fragment of BMV 1a [42] was used to detect both BMV and CCMV 1a proteins. GFP and PGK1 were detected using mouse monoclonal antibodies, clones GF28R and 22C5D8 respectively (ThermoFisher Scientific).

Bands were quantified as indicated in each figure, all quantified samples within each blot were compared in a pairwise fashion using a two-tailed Welch's t-test.

### Immunofluorescence

Yeast were prepared for confocal microscopy as previously described [64]. Goat-α-rabbit antibody conjugated to Alexa Fluor 568 (Life technologies) was used as a secondary antibody against BMV 1a antisera [42]. DNA was stained with DAPI (4′,6-diamidino-2-phenylindole, Life technologies) as a nuclear marker. The intrinsic GFP fluorescence was used to detect the GFP-2a^pol^ constructs and the Sec63-GFP ER marker [64]. Confocal images were collected on a Nikon A1. Images were processed in Fiji and are shown as an average intensity projection of five images taken across 0.8μm in the z-axis [67]. Colocalization analysis was performed in Fiji and reported as the average and standard deviation of Mander’s overlap coefficient from five cells for each condition.

### Electron microscopy

Yeast were prepared for electron microscopy by traditional chemical fixation using glutaraldehyde and paraformaldehyde followed by osmium staining as described previously [15]. High pressure freezing and freeze substitution was done according to previously described protocols [47]. Pelleted yeast were loaded into type-A planchettes and frozen in a Leica HPM-010. Freeze substitution was done in a Leica AFS II using substitution media containing: acetone, 0.25% glutaraldehyde, 0.05% UA, and 1% water. Samples were incubated in freeze substitution media at -80°C for 72 hours prior to warming to -20°C over 24 hours. Embedding was done using HM-20 (Electron Microscopy Sciences) over the course of several days at -20°C, starting with 25% HM-20 in acetone followed by 50%, 75%, and finally several washes of 100% HM-20. Each exchange was left for four hours to overnight. The resin was UV polymerized at -40°C for 48 hours followed by 12 hours of room temperature UV polymerization. All EM sections were post-stained with uranyl acetate and lead citrate [47]. Images were acquired on the Philips CM120 transmission electron microscope.
